# Successful Replantation of an Anterior Tooth in an 11-Year-Old Child: A Case Report

**DOI:** 10.7759/cureus.65794

**Published:** 2024-07-30

**Authors:** Sakshi P Kabra, Mrunali Deshkar, Nilima R Thosar, Monika Khubchandani

**Affiliations:** 1 Pediatric and Preventive Dentistry, Sharad Pawar Dental College and Hospital, Datta Meghe Institute of Higher Education and Research (Deemed to be University), Wardha, IND

**Keywords:** root canal therapy, storage media, replantation, ellis fracture, avulsion fracture

## Abstract

Avulsion occurs when the tooth is completely knocked out of its alveolar socket. The maxillary central incisors are more vulnerable to avulsion due to their prominent position in the dental arch. This case report describes a successful permanent maxillary incisor replantation in an 11-year-old child. The replanted tooth was stabilized in the socket using orthodontic wire, followed by root canal treatment and composite restoration within a two-week interval. Clinical and radiographic follow-up was done at one and six months. Successful management of an avulsed tooth requires educating the patient about different storage mediums and emergency management after an avulsion. This case report concluded that the avulsed tooth result is highly dependent on the patient's understanding of avulsion and how to approach it.

## Introduction

A severe injury known as a dental avulsion occurs when the tooth completely separates from its alveolar socket. This condition primarily has an effect on children between the ages of 8 and 11 and accounts for between 0.5% and 3% of all dental injuries [1]. Therefore, one of the elements in this age range that may increase the likelihood of oral health problems developing is a growth spurt. The way parents and dentists address avulsed teeth affects how quickly a replacement tooth heals. The state of the periodontal ligaments (PDLs) and tooth pulp is critical to the improvement of impacted teeth [2,3].

The maxillary central incisors are more vulnerable to avulsion due to their prominent position in the dental arch; as a result, they are more likely to be impacted by objects during sports, falls, or accidents. The risk of infection and root resorption after tooth extraction and replantation is a significant concern. An infection can always lead to issues that could endanger the success of the replantation. Another process that could compromise tooth integrity and durability is called root resorption, where the body's immune system mistakenly attacks the transplanted tooth root. These issues could ultimately affect the prognosis, which could significantly affect the course of treatment and the implanted tooth survival percentage [4].

Because the alveolar-dental ligament is torn during the avulsion, the remaining PDL cells on the root surface play a critical role in the survival and healing of the replanted tooth. Keeping these cells moist is necessary to preserve the health of the tooth and minimize problems; hydration facilitates the healing process and reduces the resorption phenomenon. However, if the tooth is removed from the socket for an extended period of time and the PDL dries out, the likelihood of inflammatory resorption increases. A condition characterized by the immune system attacking the tooth root compromises the long-term stability of the transplanted tooth [4]. Even with appropriate replanting care, there is still a considerable incidence of external root resorption (inflammatory and replacement types). However, over time, adhering to specific recommendations can aid in enhancing the prognosis and delaying the pace of resorption. The standards include timely reimplantation or sufficient tooth storage in an appropriate medium, careful handling to avoid further damage to the PDL, and appropriate treatment interventions, such as infection control and splinting [5].

The prognosis of the replanted tooth depends on knowing the natural history of inflammation and replacement resorption. In situations of tooth avulsion, prompt and careful care is crucial since prompt detection and management of resorption can have a substantial impact on the result. This case report describes a successful replantation of the permanent maxillary left central incisor without any complications like ankylosis or resorption.

## Case presentation

**Figure 1:**
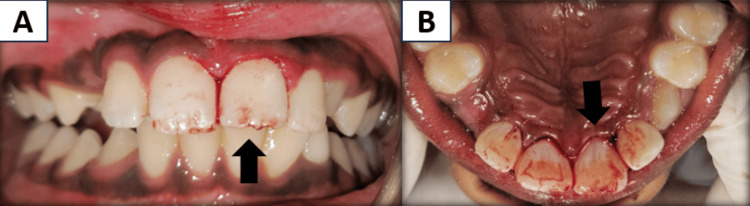
Preoperative images A and B showing immediate replantation of the maxillary left central incisor with marginal gingival laceration

An 11-year-old male patient was referred to the department of pediatric and preventive dentistry after a history of fall, causing an injury in the upper front tooth region of the jaw two hours earlier. On history recording, it was reported that the patient experienced a fall while playing on the playground, resulting in the complete separation of the maxillary left central incisor out of the socket. They immediately rushed to a private dental clinic, where the tooth was successfully reimplanted before extraoral dry time. Due to financial constraints, the patient subsequently presented to our institute for further treatment. When the patient was examined, he was conscious and showed place, time, and person orientation. There was no significant medical or systemic history present. The patient had no other signs of physical or neurological disturbances. On intra-oral soft tissue examination, a lower lip laceration was present, associated with bleeding. Localized gingival lacerations were present in 21 (Figure 1).

**Figure 2:**
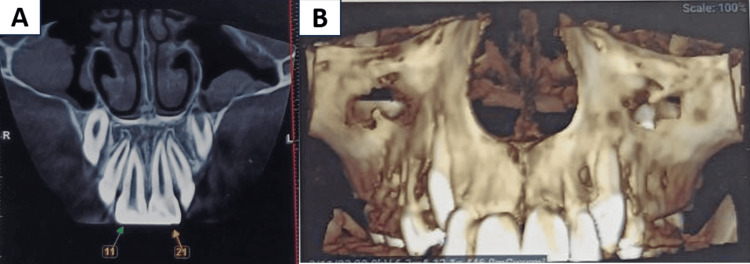
CBCT examination in images A and B revealing no fractures in the region of the maxillary anterior teeth CBCT: cone beam computed tomography

There was no clinical evidence of any other mouth injury. In the radiographic examination, cone beam CT (CBCT) was performed. The CBCT examination revealed no fractures in the region of the maxillary anterior teeth, the avulsed tooth crown was intact, the root apex was closed, and the tooth was properly reimplanted. Therefore, the case was diagnosed as an Ellis class V fracture with 21 (Figure 2).

**Figure 3:**
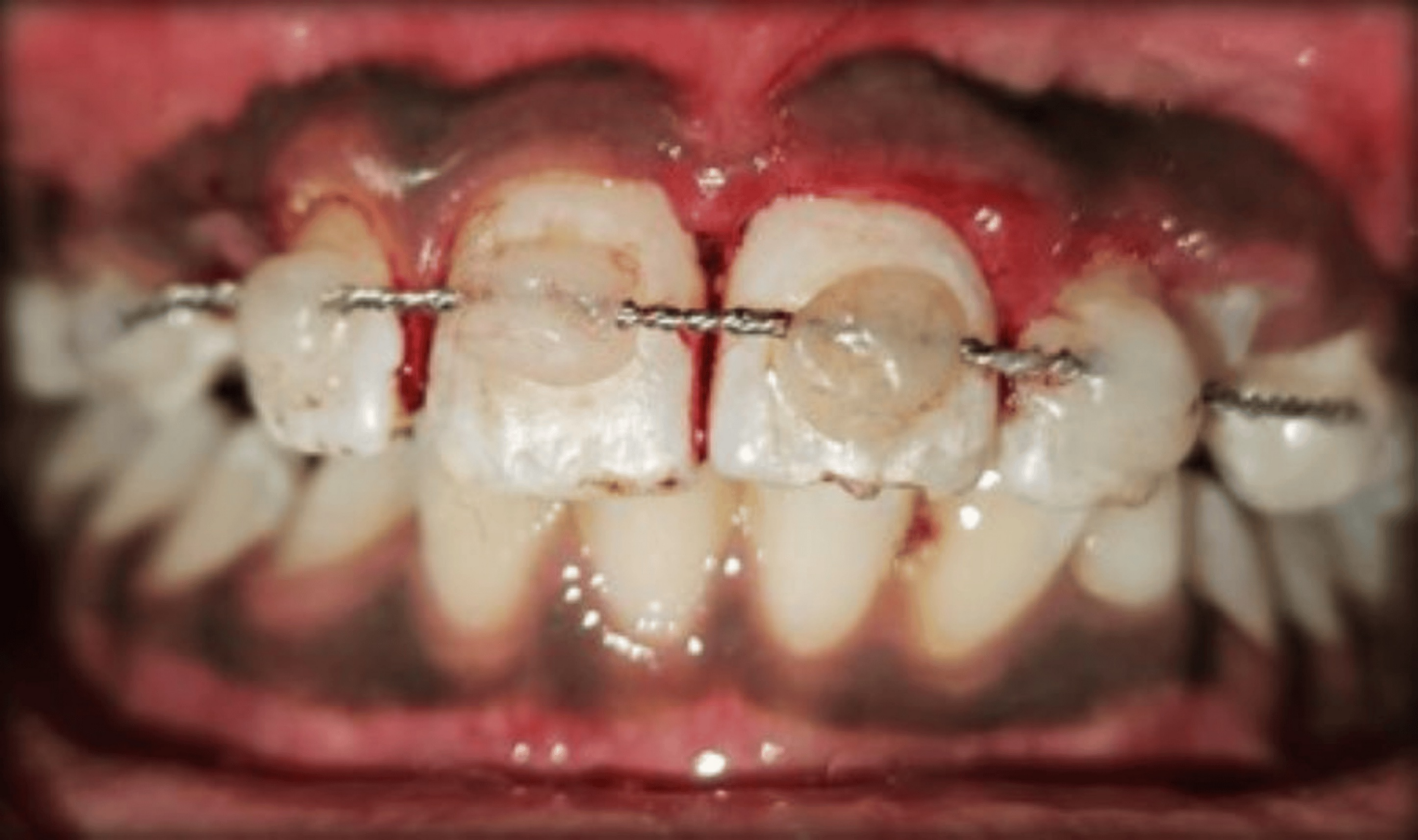
Intraoperative image showing splinting from the 13 to 23 region

Treatment was initiated by obtaining informed consent, and it was planned to splint the tooth, followed by root canal treatment. Before starting the procedure, soft tissues were carefully cleansed with saline and betadine. The replanted tooth was stabilized in the socket using an orthodontic wire of 0.4 mm in size and fixed with the help of a light-cured flowable composite from the 13 to 23 region. In the middle third of the labial surface of the crown, teeth were etched with 37% phosphoric acid for 30 seconds, followed by water rinses and air drying. Isolation was maintained using cotton rolls, and a bonding agent was applied, followed by curing for 20 seconds. An orthodontic wire was secured using flowable composite at the respective spots (Figure 3).

**Figure 4:**
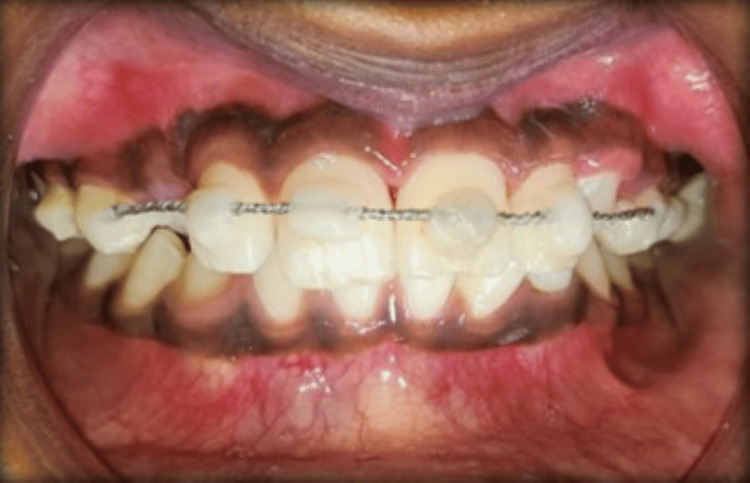
Recall after one week depicting complete resolution of mobility with 21

After splinting, antibiotics and an analgesic regime (amoxicillin 250 mg, ibuprofen 200 mg BD) were prescribed for five days. The patient was instructed to maintain a soft diet, use a soft toothbrush, and use 0.12% chlorohexidine mouthwash for oral hygiene maintenance. The patient was advised to take an anti-tetanus booster dose. The patient was recalled after one week, and mobility was completely resolved (Figure 4).

**Figure 5:**
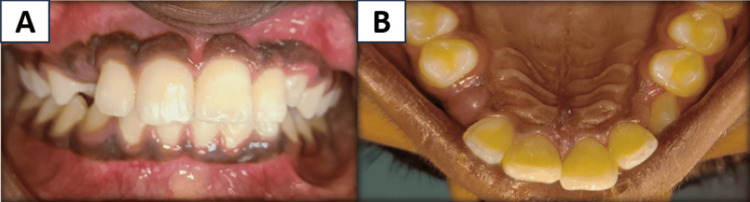
Postoperative images A and B showing the removal of the splint followed by root canal treatment and composite restoration with 21

**Figure 6:**
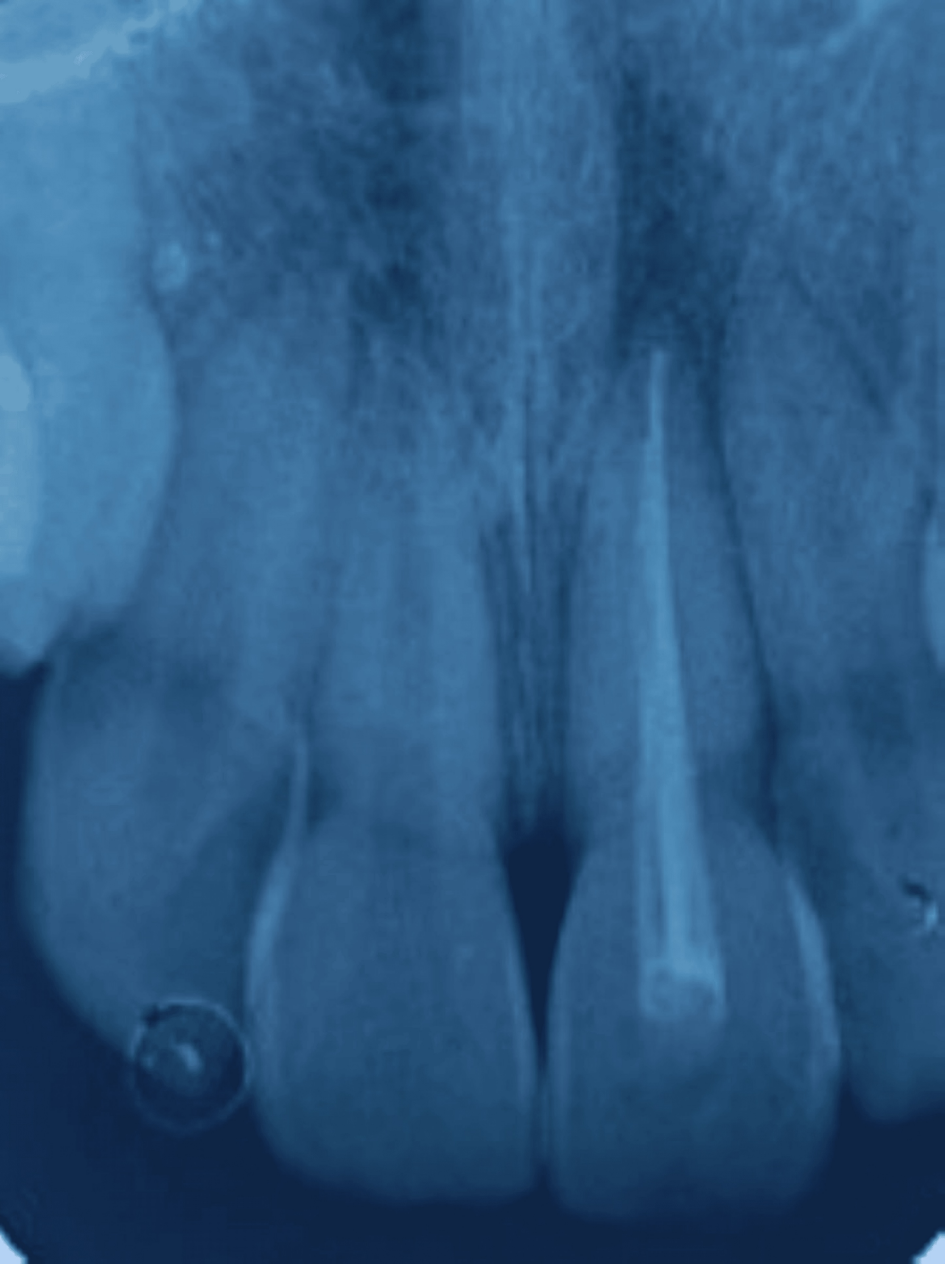
Postoperative image showing the obturation with 21

Under local anesthesia, root canal treatment was initiated without removing the splint. Access opening, biomechanical preparation, and calcium hydroxide as an intracanal medicament were placed, followed by temporary restoration with 21. After two weeks, gutta-percha obturation was done, followed by composite restoration. The splint was removed, and vitality tests were performed adjacent to the tooth to check for a positive response on electric pulp testing (Figures 5-6).

The patient was recalled after one and six months. On clinical examination, the mobility was completely resolved. Radiographic examination revealed the absence of external resorption with no signs of ankylosis with 21.

## Discussion

The potential of immediate replantation to preserve alveolar structure, function, and PDL cell viability makes it a recommended course of action for a permanent tooth that has been avulsed. In the current case, reimplantation was done of 21 before extraoral dry time.

An avulsion injury's favorable prognosis is influenced by timely intervention, meaningful treatment plans, and appropriate emergency management [6]. The general health of the patient, root maturity, storage medium, and extraoral time are the criteria that determine successful replantation [4]. With its ideal pH, growth factors, nutrients, and osmolarity, milk is the most often used and recommended storage medium for the preservation of an avulsed tooth. It is also readily available. Moreover, the epithelial growth factor, a gland secretion found in milk, promotes the growth and regeneration of Malassez epithelial cells [7]. According to a systematic review, milk is the most suitable natural storage medium due to its affordability and ease of availability, while artificial media are less effective [8].

It is crucial to stabilize the avulsed teeth because it helps the periodontal and pulp tissues repair and prevents more harm from occurring. According to a recent study, teeth can move physiologically, aiding healing and preventing ankylosis, when passive, flexible splints composed of stainless steel wire or nylon fishing line measuring 0.016" or 0.4 mm are used for a brief period of time [9]. Trope et al. recommended utilizing a composite and steel wire semi-rigid splint for seven to 10 days [10]. Two weeks is considered to be the appropriate splinting period for reimplanted avulsed teeth, depending on the length and maturity of the roots [6]. If there is an avulsion combined with cortical bone loss, the duration of splinting can be prolonged to around four to eight weeks [10]. This study used a flexible splint that was stabilized for two weeks using orthodontic wire and composite.

The International Association of Dental Traumatology recommendations state that root canal therapy ought to start no later than two weeks after the transplant. Endodontic therapy is necessary for the restored tooth because the toxins from the necrotic pulp may enter the PDL through different exit connections, accelerating the resorption process. Before replanting, it was formerly suggested to carry out extraoral root canal therapy [11]. On the other hand, intraoral root canal therapy is advised by current standards. As a result, extraoral time and related risk factors are reduced. Two weeks following replantation, endodontic therapy was started in our case, and calcium hydroxide intracanal medication was placed for a week.

A replanted tooth needs to be checked every year for at least five years after that, starting at 12 months. To rule out any difficulties, clinical and radiographic examinations must be performed at each follow-up appointment [6]. Successful management of an avulsed tooth requires educating the patient about different storage mediums and emergency management after an avulsion.

## Conclusions

Avulsed tooth results are highly dependent on the patient's understanding of avulsion and how to approach it. Because it is so crucial to the course of treatment, patient education regarding the transportation and storage of avulsed teeth must be included. Replantation, in any case, is the wisest course of action because it preserves the bone structure and function for potential future prosthetic requirements.
